# Development and Validation of Machine Models Using Natural Language Processing to Classify Substances Involved in Overdose Deaths

**DOI:** 10.1001/jamanetworkopen.2022.25593

**Published:** 2022-08-08

**Authors:** David Goodman-Meza, Chelsea L. Shover, Jesus A. Medina, Amber B. Tang, Steven Shoptaw, Alex A. T. Bui

**Affiliations:** 1 Division of Infectious Diseases, David Geffen School of Medicine at University of California, Los Angeles; 2 Division of General Internal Medicine, David Geffen School of Medicine at University of California, Los Angeles; 3 David Geffen School of Medicine at University of California, Los Angeles; 4 Department of Family Medicine, David Geffen School of Medicine at University of California, Los Angeles; 5 Medical & Imaging Informatics (MII) Group, Department of Radiological Sciences, University of California, Los Angeles

## Abstract

**Importance:**

Overdose is one of the leading causes of death in the US; however, surveillance data lag considerably from medical examiner determination of the death to reporting in national surveillance reports.

**Objective:**

To automate the classification of deaths related to substances in medical examiner data using natural language processing (NLP) and machine learning (ML).

**Design, Setting, and Participants:**

Diagnostic study comparing different natural language processing and machine learning algorithms to identify substances related to overdose in 10 health jurisdictions in the US from January 1, 2020, to December 31, 2020. Unstructured text from 35 433 medical examiner and coroners’ death records was examined.

**Exposures:**

Text from each case was manually classified to a substance that was related to the death. Three feature representation methods were used and compared: text frequency–inverse document frequency (TF-IDF), global vectors for word representations (GloVe), and concept unique identifier (CUI) embeddings. Several ML algorithms were trained and best models were selected based on F-scores. The best models were tested on a hold-out test set and results were reported with 95% CIs.

**Main Outcomes and Measures:**

Text data from death certificates were classified as any opioid, fentanyl, alcohol, cocaine, methamphetamine, heroin, prescription opioid, and an aggregate of other substances. Diagnostic metrics and 95% CIs were calculated for each combination of feature extraction method and machine learning classifier.

**Results:**

Of 35 433 death records analyzed (decedent median age, 58 years [IQR, 41-72 years]; 24 449 [69%] were male), the most common substances related to deaths included any opioid (5739 [16%]), fentanyl (4758 [13%]), alcohol (2866 [8%]), cocaine (2247 [6%]), methamphetamine (1876 [5%]), heroin (1613 [5%]), prescription opioids (1197 [3%]), and any benzodiazepine (1076 [3%]). The CUI embeddings had similar or better diagnostic metrics compared with word embeddings and TF-IDF for all substances except alcohol. ML classifiers had perfect or near perfect performance in classifying deaths related to any opioids, heroin, fentanyl, prescription opioids, methamphetamine, cocaine, and alcohol. Classification of benzodiazepines was suboptimal using all 3 feature extraction methods.

**Conclusions and Relevance:**

In this diagnostic study, NLP/ML algorithms demonstrated excellent diagnostic performance at classifying substances related to overdoses. These algorithms should be integrated into workflows to decrease the lag time in reporting overdose surveillance data.

## Key Points

**Question:**

What is the most accurate machine learning and natural language processing model to identify substances related to overdose deaths in medical examiner data?

**Findings:**

In this diagnostic study of 35 433 death records, machine learning models were able to classify with perfect or near perfect performance deaths related to any opioids, heroin, fentanyl, prescription opioids, methamphetamine, cocaine, and alcohol. Classification of benzodiazepines was suboptimal.

**Meaning:**

In this study, a natural language processing workflow was able to automate identification of substances related to overdose deaths in medical examiner data.

## Teaser

This diagnostic study assesses the use of natural language processing and machine learning to identify substances related to overdose deaths in medical examiner data.

## Introduction

Overdose deaths continue to increase in the US.^1,2^ The introduction of fentanyl in many drug markets was a substantial factor in overdose deaths since 2013. More recently, there was a shift from opioids to stimulants, with concurrent increases in methamphetamine and cocaine–related deaths. There was also an increase in overdoses related to polysubstance use, including benzodiazepines and novel compounds.^3,4^ The Centers for Disease Control and Prevention collects data from medical examiners and coroners through local health jurisdictions, summarizing overdose counts at the state and national levels on a 12-month rolling basis.^1,2^ However, these data ultimately lack local specificity, and the reporting lag makes it difficult to provide rapid responses to epidemics developing in local jurisdictions.^3,5^

Medical examiners and coroners are responsible for the first step in collection of overdose surveillance data.^6^ They determine the cause of death in cases in which an overdose is suspected and complete a corresponding death certificate. These certificates include unstructured textual data that denotes the cause of death, and in the case of an overdose, the drug involved. They are then transmitted to local jurisdictions for coding according to the *International Statistical Classification of Diseases and Related Health Problems, Tenth Revision (ICD-10)*. This coding process is done manually and is time consuming, resulting in a delay from the date of death to the correct code and additional delay from coding to the actual reporting of these deaths. This process slows the reporting of surveillance data and ensuing public health response time.

Text analysis of medical examiner entries may reveal more granular drug involvement as details may be obscured in the *ICD-10* cause of death codes used to generate national statistics.^7^ For example, both buprenorphine (a partial opioid agonist used to treat opioid use disorder) and fentanyl (a synthetic opioid that is largely illicitly manufactured) are encompassed in the same *ICD-10* code, as are tramadol, fentanyl analogs, and novel synthetic opioids such as isotonitazene. Disaggregating these data may reveal important implications for prevention ahead of national data and facilitate rapid identification of emerging drug phenomena.^8^

Natural language processing (NLP) and machine learning (ML) has the potential to automate these manual review processes. NLP is the use of computer algorithms to understand text and can be used to identify key concepts or features in text. Tied with ML, large amounts of data can be used to train models to automate tasks with high precision and accuracy.^9^ For example, Ward et al^6^ used NLP/ML to classify free-text death certificate data in Kentucky. However, this classification was limited to 1 state and identified only the presence of overdose and did not attempt to classify the contributing substance. In other applications of NLP to topics related to substance use, researchers have applied techniques to identify opioids related harms^10,11^ or overdose^12,13^ in electronic health record data.

The aim of this research was to use an automated approach to rapidly and accurately identify substances that led to death in coroners’ reports to provide more rapid surveillance data about overdoses. We assembled a database of more than 35 000 death certificates from multiple settings across the US and manually classified each of their free-text entries according to the substance involved. We compared multiple NLP and ML approaches to determine the combination of algorithms with the best diagnostic performance for identifying various substances reported within the text.

## Methods

### Data

This study entailed a cross-sectional analysis of death certificate data from multiple coroners. We obtained death certificate data from either publicly available sources or by directly requesting the data from a coroner or medical examiner. Data from January 1, 2020, to December 31, 2020, were obtained from the following counties: Cook in Illinois; Denton in Texas; Jefferson in Alabama; Johnson in Texas; Los Angeles in California; Milwaukee in Wisconsin; Parker in Texas; San Diego in California; and Tarrant in Texas. We also obtained data from the state of Connecticut. Analyses were completed in January 2022. All records provided were included in the analysis. We compiled the information into a database with the following variables: case number, county, age, gender, race, date of death, manner of death, primary cause, and secondary cause of death. The University of California at Los Angeles Institutional Review Board determined that this study was exempt from review and informed patient consent as nonhuman participant research. This study is reported following the Transparent Reporting of a Multivariable Prediction Model for Individual Prognosis or Diagnosis (TRIPOD) reporting guideline.

### Reference Standard

Two of us (C.L.S. and J.A.M.) manually classified deaths based on whether a substance was present in each case based on the accompanying text from the coroner. We compiled a dictionary of keywords to identify each substance (eTable 1 in the Supplement). A particular case could have been classified to multiple substances. We classified the text into the following categories: methamphetamine, 3,4-methylenedioxymethamphetamine, 3,4-methylenedioxyamphetamine, amphetamine, cocaine, alcohol, benzodiazepines, heroin, fentanyl, prescription opioids, any opioids, antipsychotics, antidepressant, anticonvulsants, antihistamine, muscle relaxants, barbiturates, and hallucinogens. We randomly selected 1000 records to double code and Cohen κ was calculated to rate interannotator agreement. After that step, a prespecified κ cutoff of greater than 0.80 was achieved for each group; one author (J.A.M.) coded the rest of the cases with supervision by another (C.L.S.). Only substances with at least 1000 entries were individually evaluated, and the rest were grouped as others.

### Natural Language Processing

**Figure 1.**
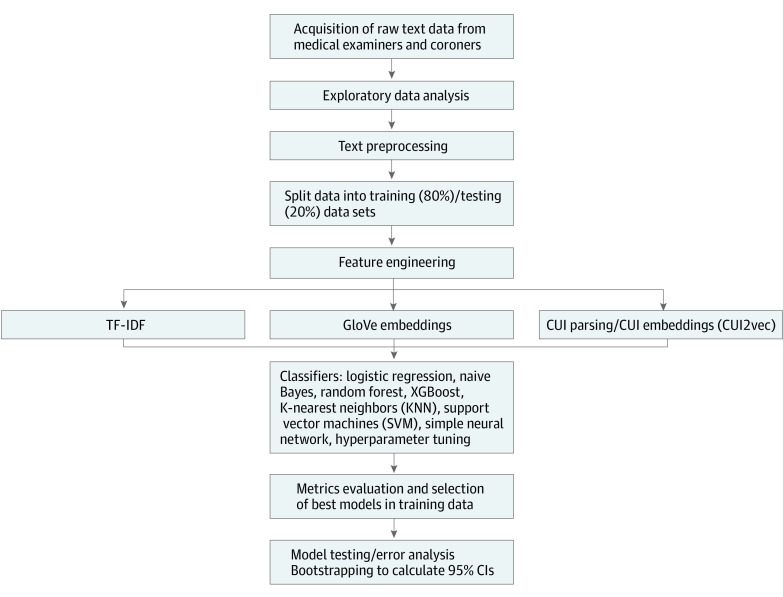
Natural Language Processing Pipeline CUI indicates concept unique identifier; GloVE, global vectors for word representations; KNN, κ-nearest neighbors; SVM, support vector machine; XGBoost, eXtreme Gradient Boosting.

Our NLP pipeline was composed of multiple stages: exploratory data analysis, data preprocessing, feature engineering, ML training and testing, and error analysis (Figure 1). During exploratory data analysis, we calculated descriptive statistics to assess the distribution of the text data and manually evaluated text entries to inform the necessary preprocessing steps. Preprocessing simplified the text before more complex modeling steps by removing entries that did not contain a description of the death (missing data), combining the primary and secondary cause of death variables, and completing basic textual formatting (removing punctuation, changing text to lower case, splitting each sentence into individual words [tokens]).

In this study, feature engineering entailed creating numeric representations from the source textual data. We compared 3 methods for feature engineering: text frequency–inverse document frequency (TF-IDF), word embeddings, and embeddings of concept unique identifiers (CUIs). The TF-IDF is a frequency based numeric representation of each word calculated as the product of the TF (number of times a word appears in each observation) and IDF. The IDF is calculated as the log of the number of documents divided by the number of documents that contain the word in question. In contrast, word embeddings are numeric representations of words in multidimensional spaces that are obtained from pretrained models. In our word embeddings models, we used global vectors for word representations (GloVe), a model pretrained on text from Wikipedia and the Gigaword 5 corpus (newswire text data).^14^ We used a version of GloVe that is composed of 6 billion tokens and 100 dimensions.^15,16^ In addition, CUIs are unique codes assigned to each concept in a particular terminology, in this case medical. We used the *scispacy* framework^17^ to link text in the data set to their particular CUI in the National Library of Medicine Unified Medical Language System.^18^ Then, we matched each CUI to their respective embedding. Similar to word embeddings, vector embeddings are numeric representations of CUIs in multidimensional spaces obtained from pretrained models. We used *CUI2vec*, a model pretrained on text from a collection of 20 million clinical notes and 1.7 million biomedical journal articles.^19^ The *CUI2vec* provided embeddings for 109 053 unique CUIs and 500 dimensions for each CUI. The CUI2vec embeddings were filtered to include only those with a semantic class of *organic chemical*.

### Machine Learning Classifiers

Next, we evaluated multiple ML classification models that included logistic regression, naïve Bayes algorithm, random forest, XGBoost, κ-nearest neighbors, support vector machines, and a single-layer neural network. Separate binary classifiers were trained and tested for each substance evaluated. For each substance, we split the data 80% for training and 20% for testing and final evaluation (ie, hold-out test set) stratified by said substance. We trained all classifiers on the training split using 10-fold cross-validation. We tuned hyperparameters for models such as random forest, XGBoost, κ-nearest neighbors, support vector machines, and neural networks based on a grid search method. In this strategy, we trained a model with an initial set of hyperparameters, and then reran the same model with values around the initial values, and subsequently around the value of the previous step. We used the model and combination of hyperparameters with the best F-score (harmonic mean of positive predictive value and sensitivity) for testing.

### Statistical Analysis

We calculated final diagnostic metrics for each substance and model on the held-out-test set (20% of data). Final diagnostic metrics included F-score, accuracy, κ, sensitivity (recall), specificity, positive predictive value (PPV; precision), negative predictive value, and area under the receiver operating curve. We calculated 95% CIs by bootstrapping by resampling the testing set with replacement 1000 times and calculating diagnostic metrics for each resample. We reported the 2.5th percentile as the lower end of the CI and 97.5th percentile as the upper end of the CI and the 50th percentile as the mean. We created confusion matrixes to identify the number of false positives, true positives, false negatives, and true negatives. Two of us (A.T. and D.G.M.) manually evaluated the false negative and false positive cases to identify the reasons for incorrect classification. To attempt to identify keywords that models used for their predictions, we plotted feature importance plots based on TF-IDF and logistic regression. All analyses were performed in R version 4.0.2 (R Foundation for Statistical Computing) using the *tidymodels* framework on an Amazon Web Server.

## Results

### Descriptive

**Figure 2.**
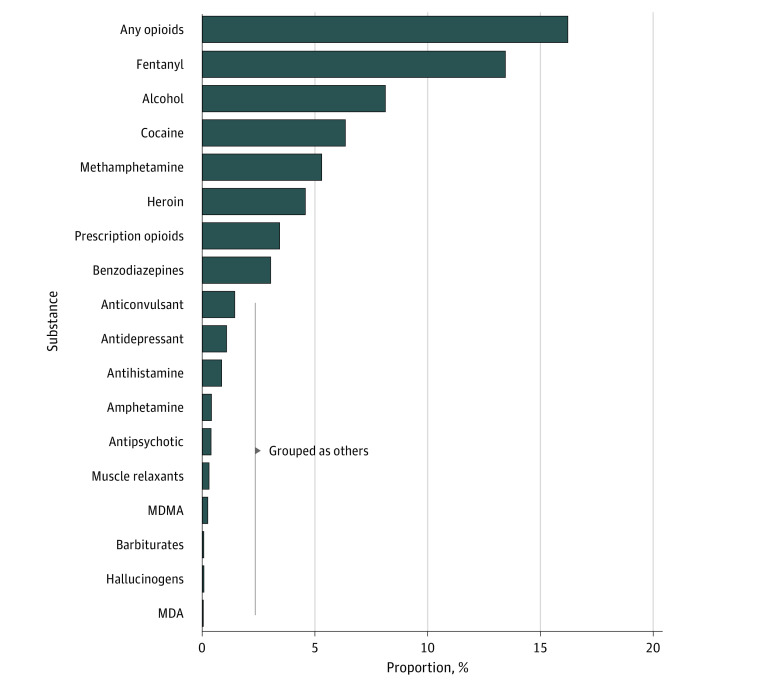
Substances Identified in Overdoses From Medical Examiner Data MDA indicates 3,4-methylenedioxyamphetamine; MDMA, 3,4-methylenedioxymethamphetamine.

The initial data set included 35 698 cases. We excluded 265 cases because of missing textual data, resulting in a final data set of 35 433 cases. The decedent median age was 58 years (IQR, 41-72 years) and 24 449 (69%) were male. The jurisdictions that provided the most cases were Cook County (45%), Los Angeles County (32%), and San Diego County (8%). The median number of characters per text for each case was 59 (range, 3 to 331). The median number of words per text was 7 (range, 1 to 38). The number of substances or groups of substances classified were 0 in 26 695 cases (75%); 1 in 2635 cases (7%); 2 in 1401 cases (4%); 3 in 2218 cases (6%); 4 in 1364 cases (4%); 5 in 659 cases (2%); 6 in 301 cases (1%); 7 in 113 cases (<1%); 8 in 41 cases (<1%); and 9 in 6 cases (<1%). The substances or groups of substances identified to be related to a death are shown in Figure 2 and include any opioid (5739 [16%]), fentanyl (4758 [13%]), alcohol (2866 [8%]), cocaine (2247 [6%]), methamphetamine (1876 [5%]), heroin (1613 [5%]), prescription opioids (1197 [3%]), and any benzodiazepine (1076 [3%]). Substances with a count below a cutoff of 1000 (eg, 3,4-methylenedioxymethamphetamine, 3,4-methylenedioxyamphetamine, amphetamine, antipsychotics, antidepressant, anticonvulsants, antihistamine, muscle relaxants, barbiturates, and hallucinogens) were grouped as others. eTable 2 in the Supplement presents a matrix of co-occurrence of substances involved in deaths.

### Diagnostic Metrics

Table 1 presents the F-score results from the 10-fold cross-validation performed on the hold-out test set. Models using both TF-IDF, word embeddings, and CUI embeddings performed almost perfectly in identifying any opioids, heroin, fentanyl, methamphetamines, and cocaine. Notably, classification of prescription opioids was suboptimal using TF-IDF (F-score, 0.571) and word embeddings (F-score, 0.554); whereas CUI embeddings performed nearly perfectly for prescription opioids (F-score, 0.996). Benzodiazepines performance was suboptimal across all 3 feature representations (F-scores: CUI embeddings, 0.902; TF-IDF, 0.795; word embeddings, 0.662). For alcohol, TF-IDF had and F-score of 0.972 and word embeddings had an F-score of 0.956, whereas CUI embeddings had a lower F-score of 0.852.

**Table 1.** Top 3 Models by Substance in 10-Fold Cross-Validation of Training Data Set

Substance	TF-IDF			Word embeddings (GloVe)^a^			CUI2Vec embeddings^b^		
	Model	Mean		Model	Mean		Model	Mean	
		F-score	SE		F-score	SE		F-score	SE
Any opioid	XGBoost^c^	0.969	0.002	SVM^c^	0.970	0.002	SVM^c^	0.992	0.001
	Random forest^c^	0.969	0.001	Neural network	0.967	0.003	XGBoost	0.989	0.001
	Neural network	0.968	0.002	XGBoost	0.965	0.003	Random forest	0.987	0.001
Heroin	Logistic regression^c^	1.000	0.000	Logistic regression^c^	1.000	0.000	Logistic regression^c^	1.000	0.000
	Random forest^c^	1.000	0.000	SVM^c^	1.000	0.000	SVM^c^	1.000	0.000
	XGBoost^c^	1.000	0.000	Neural network	0.999	0.000	XGBoost	0.996	0.002
Fentanyl	Random forest^c^	1.000	0.000	SVM^c^	1.000	0.000	SVM^c^	1.000	0.000
	XGBoost^c^	1.000	0.000	Neural network^c^	1.000	0.000	Neural network^c^	1.000	0.000
	Logistic regression	0.999	0.000	Logistic regression^c^	1.000	0.000	XGBoost	0.999	0.000
Prescription opioid	XGBoost^c^	0.561	0.015	XGBoost^c^	0.554	0.015	SVM^c^	0.996	0.002
	Random forest	0.558	0.015	Random forest	0.514	0.016	Neural network	0.989	0.002
	Logistic regression	0.545	0.015	SVM	0.510	0.012	Logistic regression	0.985	0.002
Methamphetamine	Logistic regression^c^	1.000	0.000	SVM^c^	0.999	0.000	SVM^c^	0.998	0.001
	Random forest^c^	1.000	0.000	Neural network	0.997	0.002	Logistic regression	0.987	0.005
	XGBoost^c^	1.000	0.000	Logistic regression	0.997	0.001	XGBoost	0.986	0.001
Cocaine	Logistic regression^c^	1.000	0.000	Logistic regression^c^	1.000	0.000	Logistic regression^c^	1.000	0.000
	Random forest^c^	1.000	0.000	SVM^c^	1.000	0.000	SVM^c^	1.000	0.000
	XGBoost^c^	1.000	0.000	SVM	0.999	0.000	Neural network	0.998	0.001
Benzodiazepine	Random forest^c^	0.671	0.013	Neural network^c^	0.662	0.011	Neural network^c^	0.902	0.009
	XGBoost	0.666	0.015	SVM	0.645	0.016	SVM^c^	0.902	0.009
	Neural network	0.657	0.013	XGBoost	0.637	0.014	XGBoost	0.867	0.01
Alcohol	Random forest^c^	0.974	0.003	SVM^c^	0.956	0.002	XGBoost^c^	0.852	0.005
	XGBoost	0.974	0.003	Neural network	0.951	0.003	Random forest^c^	0.852	0.005
	Neural network	0.973	0.003	XGBoost	0.948	0.002	SVM	0.851	0.005
Other	XGBoost	0.812	0.005	Neural network^c^	0.806	0.005	SVM^c^	0.968	0.003
	Random forest	0.811	0.004	XGBoost	0.804	0.003	Logistic regression	0.953	0.006
	Neural network	0.807	0.004	Random forest	0.772	0.004	XGBoost	0.941	0.004

Abbreviations: CUI, concept unique identifier; GloVe, global vectors for word representations; SVM, support vector machine; TF-IDF, term frequency–inverse document frequency.

^a^ GloVe with 6 billion tokens and 100 dimensions was used in this analysis.

^b^ CUI2vec with 109 053 tokens and 500 dimensions was used in this analysis.

^c^ The best performing models based on the mean F-score of 10-fold cross-validation.

eTable 3 in the Supplement displays the bootstrapped diagnostic metrics for the best models using TF-IDF. Final models had high F-scores for any opioids (F-score, 0.969; 95% CI, 0.959-0.979), heroin (F-score, 1.00; 95% CI, 1.00-1.00), fentanyl (F-score, 0.999; 95% CI, 0.998-1.00), methamphetamine (F-score, 0.992; 95% CI, 0.979-0.997), cocaine (F-score, 0.999; 95% CI, 0.997-1.00), and alcohol (F-score, 0.968; 95% CI, 0.953-0.980). The TF-IDF models were suboptimal at identifying prescription opioids (F-score, 0.308; 95% CI, 0.211-0.468), benzodiazepines (F-score, 0.771; 95% CI, 0.716-0.826), and others (F-score, 0.777; 95% CI, 0.743-0.808).

eTable 4 in the Supplement displays the bootstrapped diagnostic metrics from the hold-out test set for the best selected models using word embeddings. These models performed with a high F-score for classifying a death as related to any opioid (F-score, 0.966; 95% CI, 0.956-0.976), heroin (F-score, 1.00; 95% CI, 1.00-1.00), fentanyl (F-score, 0.999; 95% CI, 0.998-1.00), methamphetamine (F-score, 0.998; 95% CI, 0.993-1.00), cocaine (F-score, 0.999; 95% CI, 0.997-1.00), and alcohol (F-score, 0.942; 95% CI, 0.924-0.960). Suboptimal classification occurred for prescription opioids (F-score, 0.378; 95% CI, 0.205-0.537), benzodiazepines (F-score, 0.771; 95% CI, 0.716-0.826), and others (F-score, 0.750; 95% CI, 0.715-0.785).

Last, Table 2 displays the bootstrapped diagnostic metrics from the hold-out test set for the best selected models using CUI embeddings. Models had excellent performance at classifying deaths related to any opioid (F-score, 0.989; 95% CI, 0.982-0.994), heroin (F-score, 1.00; 95% CI, 1.00-1.00), fentanyl (F-score, 0.999; 95% CI, 0.998-1.00), prescription opioids (F-score, 0.977; 95% CI, 0.941-1.00), methamphetamine (F-score, 0.995; 95% CI, 0.989-1.00), cocaine (F-score, 1.00; 95% CI, 1.00-1.00), and others (F-score, 0.942; 95% CI, 0.924-0.960). Again, suboptimal classification occurred for benzodiazepines (F-score, 0.840; 95% CI, 0.788-0.889), and alcohol (F-score, 0.854; 95% CI, 0.828-0.880).

**Table 2.** Bootstrapped Diagnostic Metrics and Best Performing Models in Test Data Set (N = 7087) Using CUI2Vec as Feature Representations^a^

Metric	Mean (95% CI)^b^								
	Any opioid	Heroin	Fentanyl	Prescription opioid	Methamphetamine	Cocaine	Benzodiazepine	Alcohol	Other
F-score	0.989 (0.982-0.994)	1.00 (1.00-1.00)	0.999 (0.997-1.00)	0.977 (0.941-1.00)	0.995 (0.989-1.00)	1.00 (1.00-1.00)	0.840 (0.788-0.889)	0.854 (0.828-0.880)	0.950 (0.933-0.965)
Accuracy	0.996 (0.994-0.998)	1.00 (1.00-1.00)	1.00 (0.999-1.00)	0.998 (0.996-1.00)	0.999 (0.999-1.00)	1.00 (1.00-1.00)	0.988 (0.967-0.993)	0.979 (0.975-0.983)	0.992 (0.990-0.995)
κ	0.986 (0.979-0.993)	1.00 (1.00-1.00)	0.999 (0.997-1.00)	0.977 (0.939-1.00)	0.995 (0.988-1.00)	1.00 (1.00-1.00)	0.722 (0-0.885)	0.843 (0.815-0.871)	0.945 (0.928-0.962)
Sensitivity (recall)	0.98 (0.970-0.989)	1.00 (1.00-1.00)	0.999 (0.997-1.00)	0.971 (0.931-1.00)	0.993 (0.98-1.00)	1.00 (1.00-1.00)	0.658 (0-0.829)	0.749 (0.709-0.787)	0.912 (0.885-0.938)
Specificity	1 (0.998-1.00)	1.00 (1.00-1.00)	1.00 (1.00-1.00)	0.999 (0.998-1.00)	1.00 (1.00-1.00)	1.00 (1.00-1.00)	0.999 (0.996-1.00)	1.00 (0.997-1.00)	0.999 (0.998-1.00)
Positive predictive value (precision)	0.998 (0.988-1.00)	1.00 (1.00-1.00)	1.00 (0.997-1.00)	0.984 (0.931-1.00)	0.997 (0.992-1.00)	1.00 (1.00-1.00)	0.940 (0.873-0.986)	0.994 (0.955-1.00)	0.99 (0.973-1.00)
Negative predictive value	0.996 (0.994-0.998)	1.00 (1.00-1.00)	1.00 (1.00-1.00)	0.999 (0.998-1.00)	1.00 (0.999-1.00)	1.00 (1.00-1.00)	0.989 (0.967-0.995)	0.978 (0.974-0.982)	0.992 (0.99-0.995)
AUROC	0.994 (0.988-0.999)	1.00 (1.00-1.00)	1.00 (0.997-1.00)	0.987 (0.965-1.00)	0.997 (0.986-1.00)	1.00 (1.00-1.00)	0.940 (0.895-0.978)	0.901 (0.883-0.918)	0.981 (0.956-0.995)

Abbreviation: AUROC, area under the receiver operating curve.

^a^ CUI2vec with 109 053 tokens and 500 dimensions was used in this analysis.

^b^ Values are means of 1000 resamples bootstrapping procedure, values in parenthesis are lower and upper bounds of 95% percentiles for the bootstrapping procedure.

### Error Analysis and Interpretability

We present confusion matrices for the true positive, false positive, true negative and false negative values for each substance derived from the analysis in the held-out test set (eTables 5-13 in the Supplement). We also completed a subsequent error analysis (eTables 14-19 in the Supplement), in which we manually identified mistakes made by the models. eFigures 1-9 in the Supplement show feature importance plots for each substance or group of substances.

## Discussion

In this diagnostic study, we present results for the use of NLP for feature extraction and ML to classify specific substances related to overdose deaths. We found that for most substances evaluated, the performance of these algorithms was perfect or near perfect. These models could be used to automate classification of unstructured free-text, thus avoiding the manual and time-consuming process of individually reading each entry and classifying them to a specific substance. However, more work is needed for rapid identification of certain substances, such as benzodiazepines, because the models studied did not have a high diagnostic performance. However, they were able to reliably exclude (ie, high negative predictive value) cases that did not contain the substance in question. This ability could help exclude a vast number of cases, concentrating manual review on cases classified as positive. Ultimately, adoption of NLP/ML tools such as the ones developed and tested in this study could provide rapid results for policy makers, clinicians, and harm reduction agencies to respond appropriately in their respective areas.

We build on the successful implementation of NLP from previous work by colleagues in the field.^6^ Whereas prior detection has been concentrated on identification of overdose alone, we extended NLP to identify the substance associated with overdose cases. Additional strengths of the present study include the large number of cases available for training and testing, the multitude and specificity of substances that we classified. We have also provided our data and code as an open-source repository for future researchers to build on for further improvement. Further validation will be needed to verify the external validity of these models to data from jurisdictions outside of this initial evaluation.

Excellent performance was shown for multiple substances, including any opioid, heroin, fentanyl, methamphetamine, cocaine, and alcohol using models for general text (word embeddings or TF-IDF). Yet for prescription opioids and benzodiazepines, there was a considerable performance gap. The substances that performed well in the models may have done so because of a relatively small number of words commonly used in their identification (eg, heroin, fentanyl, methamphetamine, and cocaine). They may also have performed well because of the large number of data entries available for training (eg, any opioid and fentanyl). Of the substances we included in the model, prescription opioids and benzodiazepines had the smallest number of data entries. In addition, owing to the large number of keywords for both groups, models may have had difficulty identifying uncommon terms in the training data. We expect that with more data, model performance would improve. Other factors complicating model predictions of prescription opioids and benzodiazepines may include difficulty identifying nuances between prescription opioids (eg, oxycodone and hydrocodone) and illicit opioids (eg, heroin and fentanyl), or the coexistence of multiple other substances (deaths due to polysubstance use). Furthermore, more common substances (eg, alcohol, cocaine, and heroin) may be part of the general lexicon and thus identifiable by general embeddings such as GloVe. However, novel or less commonly used substances (including the diversity of prescription opioids and benzodiazepines) are unlikely to appear in the general text that these models were trained on.

When we tested a feature extraction method specific to medical terminology (CUI embeddings), performance improved across most substances, most notably prescription opioids and benzodiazepines. However, some identification errors occurred owing to the lack of a specific CUI for certain substances. For instance, alprazolam coded to C0002333, but flualprazolam did not map to a specific entry in *scispacy* and was therefore unable to be identified in the model. This lack may lead to problems when encountering novel substances without specific concept identifiers that are involved in overdoses. An example of this in our analysis was carfentanyl, a novel fentanyl analog was not captured either.^20^ Over time, an iterative process of error analysis and retraining will be necessary to ensure ongoing accuracy.

Future directions include the use of more sophisticated models such as Deep Bidirectional Transformers for Language Understanding,^21^ more specific medical^22^ or clinical^23^ models for NLP, or deep learning methods such as convolutional neural networks. For more straightforward identification of substances, the simpler models we opted for here yielded excellent classification results. However, for the substances in which performance was suboptimal, these approaches should be further explored.

### Limitations

This study has limitations. A main limitation of this work includes the inability to train models for less common substances in our data set, ranging from generalized groups of medications (eg, anticonvulsants) to individual drugs (eg, 3,4-methylenedioxymethamphetamine). As the models rely on a large volume of training cases to learn and make predictions, they would likely not be reliable in the automatic identification of emerging trends. However, other clustering or unsupervised models could be used to identify emerging trends and should be explored in future tasks. Over time, the failure of these models may also be an indicator of how we think about data set shift in this space and emergent causes that are not within the common knowledge space. In addition, it is unknown how these models may generalize to other areas of the country as the models were trained heavily from data from 3 urban centers.

## Conclusions

Rapid and accurate data are necessary to adequately implement policies and develop interventions to address the increasing overdose crisis in the US. In this analysis, we found that NLP and ML are tools that may provide excellent results for rapid classification of unstructured text data produced by medical examiners and coroners. The NLP tools such as these should be integrated in data surveillance workflows to increase rapid dissemination of data to the public, researchers, and policy makers.

## Notes

Supplement. **eTable 1.** Classifications and Keywords of Substances Related to Overdoses **eTable 2.** Co-occurrence of Substances Involved in Overdose Deaths **eTable 3.** Bootstrapped diagnostic Metrics and 95% Confidence Intervals of Best Performing Models in Test Dataset (N=7,087) Using TF-IDF as Feature Representations **eTable 4.** Bootstrapped Diagnostic Metrics and 95% Confidence Intervals of Best Performing Models in Test Dataset (N=7,087) Using Word Embedding (GloVe) as Feature Representations **eTable 5.** Confusion Matrix for Any Opioids **eTable 6.** Confusion Matrix for Heroin **eTable 7.** Confusion Matrix for Fentanyl **eTable 8.** Confusion Matrix for Prescription Opioids **eTable 9.** Confusion Matrix for Methamphetamine **eTable 10.** Confusion Matrix for Cocaine **eTable 11.** Confusion Matrix for Benzodiazepines **eTable 12.** Confusion Matrix for Alcohol **eTable 13.** Confusion Matrix for Others **eTable 14.** Error Analysis for Any Opioids **eTable 15.** Error Analysis for Fentanyl **eTable 16.** Error Analysis for Prescription Opioids **eTable 17.** Error Analysis for Benzodiazepines **eTable 18.** Error Analysis for Alcohol **eTable 19.** Error Analysis for “Other” Substances **eFigure 1.** Variable Importance Plot for Predicting Category “Any Opioids” **eFigure 2.** Variable Importance Plot for Predicting Category “Heroin” **eFigure 3.** Variable Importance Plot for Predicting Category “Fentanyl” **eFigure 4.** Variable Importance Plot for Predicting Category “Prescription Opioids” **eFigure 5.** Variable Importance Plot for Predicting Category “Methamphetamine” **eFigure 6.** Variable Importance Plot for Predicting Category “Cocaine” **eFigure 7.** Variable Importance Plot for Predicting Category “Benzodiazepines” **eFigure 8.** Variable Importance Plot for Predicting Category “Alcohol” **eFigure 9.** Variable Importance Plot for Predicting Category “Others”

## References

[1] Friedman J, Akre S. COVID-19 and the drug overdose crisis: uncovering the deadliest months in the United States, January–July 2020. Am J Public Health. 2021;111(7):1284-1291. doi:10.2105/AJPH.2021.306256 33856885 PMC8493145

[2] Ahmad F, Rossen LM, Sutton P. Provisional drug overdose death counts. National Center for Health Statistics. Published 2021. Accessed July 7, 2022. https://www.cdc.gov/nchs/nvss/vsrr/drug-overdose-data.htm

[3] Dasgupta N, Beletsky L, Ciccarone D. Opioid crisis: no easy fix to its social and economic determinants. Am J Public Health. 2018;108(2):182-186. doi:10.2105/AJPH.2017.304187 29267060 PMC5846593

[4] Mattson CL, Tanz LJ, Quinn K, Kariisa M, Patel P, Davis NL. Trends and geographic patterns in drug and synthetic opioid overdose deaths - United States, 2013-2019. MMWR Morb Mortal Wkly Rep. 2021;70(6):202-207. doi:10.15585/mmwr.mm7006a4 33571180 PMC7877587

[5] Shover CL, Falasinnu TO, Dwyer CL, . Steep increases in fentanyl-related mortality west of the Mississippi River: recent evidence from county and state surveillance. Drug Alcohol Depend. 2020;216:108314. doi:10.1016/j.drugalcdep.2020.108314 33038637 PMC7521591

[6] Ward PJ, Rock PJ, Slavova S, Young AM, Bunn TL, Kavuluru R. Enhancing timeliness of drug overdose mortality surveillance: a machine learning approach. PLoS One. 2019;14(10):e0223318. doi:10.1371/journal.pone.0223318 31618226 PMC6795484

[7] Shiue KY, Austin AE, Proescholdbell S, Cox ME, Aurelius M, Naumann RB. Literal text analysis of poly-class and polydrug overdose deaths in North Carolina, 2015-2019. Drug Alcohol Depend. 2021;228:109048. doi:10.1016/j.drugalcdep.2021.109048 34601275 PMC12925364

[8] Shover CL, Falasinnu TO, Freedman RB, Humphreys K. Emerging characteristics of isotonitazene-involved overdose deaths: a case-control study. J Addict Med. 2021;15(5):429-431. doi:10.1097/ADM.0000000000000775 33234804 PMC8141068

[9] Nadkarni PM, Ohno-Machado L, Chapman WW. Natural language processing: an introduction. J Am Med Inform Assoc. 2011;18(5):544-551. doi:10.1136/amiajnl-2011-000464 21846786 PMC3168328

[10] Badger J, LaRose E, Mayer J, Bashiri F, Page D, Peissig P. Machine learning for phenotyping opioid overdose events. J Biomed Inform. 2019;94:103185. doi:10.1016/j.jbi.2019.103185 31028874 PMC6622451

[11] Lingeman JM, Wang P, Becker W, Yu H. Detecting opioid-related aberrant behavior using natural language processing. AMIA Annu Symp Proc. 2018;2017:1179-1185. 29854186 PMC5977697

[12] Green CA, Perrin NA, Hazlehurst B, . Identifying and classifying opioid-related overdoses: a validation study. Pharmacoepidemiol Drug Saf. 2019;28(8):1127-1137. doi:10.1002/pds.4772 31020755 PMC6767606

[13] Hazlehurst B, Green CA, Perrin NA, . Using natural language processing of clinical text to enhance identification of opioid-related overdoses in electronic health records data. Pharmacoepidemiol Drug Saf. 2019;28(8):1143-1151. doi:10.1002/pds.4810 31218780 PMC6772185

[14] Parker R, Graff D, Kong J, Chen K, Maeda K. English gigaword fifth edition. Linguistic Data Consortium. June 17, 2011. Accessed July 6,7, 2022. doi:10.35111/wk4f-qt80

[15] Wang Y, Liu S, Afzal N, . A comparison of word embeddings for the biomedical natural language processing. J Biomed Inform. 2018;87:12-20. doi:10.1016/j.jbi.2018.09.008 30217670 PMC6585427

[16] Pennington J, Socher R, Manning C. GloVe: global vectors for word representation. In: *Proceedings of the 2014 Conference on Empirical Methods in Natural Language Processing (EMNLP)*. Association for Computational Linguistics; 2014:1532-1543.

[17] Neumann M, King D, Beltagy I, Ammar W. ScispaCy: Fast and Robust Models for Biomedical Natural Language Processing. arxiv. Preprint posted online October 9, 2019. doi:10.18653/v1/W19-5034

[18] Bodenreider O. The Unified Medical Language System (UMLS): integrating biomedical terminology. Nucleic Acids Res. 2004;32(Database issue):D267-D270. doi:10.1093/nar/gkh061 14681409 PMC308795

[19] Beam AL, Kompa B, Schmaltz A, . Clinical concept embeddings learned from massive sources of multimodal medical data. Pac Symp Biocomput. 2020;25:295-306. 31797605 PMC6922053

[20] O’Donnell J, Tanz LJ, Gladden RM, Davis NL, Bitting J. Trends in and characteristics of drug overdose deaths involving illicitly manufactured fentanyls - United States, 2019-2020. MMWR Morb Mortal Wkly Rep. 2021;70(50):1740-1746. doi:10.15585/mmwr.mm7050e3 34914673 PMC8675656

[21] Devlin J, Chang M-W, Lee K, Toutanova K. BERT: pre-training of deep bidirectional transformers for language understanding. arXiv. Preprint posted online May 24, 2019. doi:10.48550/arXiv.1810.04805

[22] Lee J, Yoon W, Kim S, . BioBERT: a pre-trained biomedical language representation model for biomedical text mining. Bioinformatics. 2020;36(4):1234-1240. 31501885 10.1093/bioinformatics/btz682 PMC7703786

[23] Alsentzer E, Murphy JR, Boag W, . Publicly available clinical BERT embeddings. arXiv. Preprint posted online June 20, 2019. doi:10.48550/arXiv.1904.03323

