# Supplementary material for: Development and Validation of Machine Models Using Natural Language Processing to Classify Substances Involved in Overdose Deaths
Source: JAMA Netw Open. 2022 Aug 8;5(8):e2225593. doi: 10.1001/jamanetworkopen.2022.25593 (PMC9361079; doi:10.1001/jamanetworkopen.2022.25593)

Supplemental Online Content

Goodman-Meza D, Shover CL, Medina JA, Tang AB, Shoptaw S, Bui AAT. Development and validation of machine models using natural language processing to classify substances involved in overdose deaths. *JAMA Netw Open.* 2022;5(8):e2225593. doi:10.1001/jamanetworkopen.2022.25593

eTable 1. Classifications and Keywords of Substances Related to Overdoses

eTable 2. Co-occurrence of Substances Involved in Overdose Deaths

eTable 3. Bootstrapped Diagnostic Metrics and 95% Confidence Intervals of Best Performing Models in Test Dataset (N = 7,087) Using TF-IDF as Feature Representations

eTable 4. Bootstrapped Diagnostic Metrics and 95% Confidence Intervals of Best Performing Models in Test Dataset (N = 7,087) Using Word Embedding (GloVe) as Feature Representations

eTable 5. Confusion Matrix for Any Opioids

eTable 6. Confusion Matrix for Heroin

eTable 7. Confusion Matrix for Fentanyl

eTable 8. Confusion Matrix for Prescription Opioids

eTable 9. Confusion Matrix for Methamphetamine

eTable 10. Confusion Matrix for Cocaine

eTable 11. Confusion Matrix for Benzodiazepines

eTable 12. Confusion Matrix for Alcohol

eTable 13. Confusion Matrix for Others

eTable 14. Error Analysis for Any Opioids

eTable 15. Error Analysis for Fentanyl

eTable 16. Error Analysis for Prescription Opioids

© 2022 Goodman-Meza D et al. *JAMA Network Open*.

eTable 17. Error Analysis for Benzodiazepines

eTable 18. Error Analysis for Alcohol

eTable 19. Error Analysis for “Other” Substances

eFigure 1. Variable Importance Plot for Predicting Category “Any Opioids”

eFigure 2. Variable Importance Plot for Predicting Category “Heroin”

eFigure 3. Variable Importance Plot for Predicting Category “Fentanyl”

eFigure 4. Variable Importance Plot for Predicting Category “Prescription Opioids”

eFigure 5. Variable Importance Plot for Predicting Category “Methamphetamine”

eFigure 6. Variable Importance Plot for Predicting Category “Cocaine”

eFigure 7. Variable Importance Plot for Predicting Category “Benzodiazepines”

eFigure 8. Variable Importance Plot for Predicting Category “Alcohol”

eFigure 9. Variable Importance Plot for Predicting Category “Others”

This supplemental material has been provided by the authors to give readers additional information about their work.

© 2022 Goodman-Meza D et al. *JAMA Network Open*.

eTable 1. Classifications and keywords of substances related to overdoses.

Classification	Keywords
**Alcohol**	Alcohol, ethanol, ethanolism
**Amphetamine**	Amphetamine (only instances were amphetamine without methamphetamine were counted in this category)
**Anticonvulsants**	Carbamazepine, clobazam, oxcarbazepine, diazepam, ethosuxamide, phenytoin, gabapentin, lacosamide, levetiracetam, phenobarbital, pregabalin, lamotrigine, topiramate, valproate, valproic acid, zonisamide
**Anti-depressants**	Citalopram, fluoxetine, fluvoxamine, paroxetine, sertraline, buproprion, venlafaxine, duloxetine, desvenlafasxine, levomilnacipran, imipramine, desipramine, nortriptyline, doxepin, trimipramine, amoxapine, protriptyline, trazodone, mirtazapine
**Antihistamines**	Diphenydramine, cetirizine, chlorpheniramine, fexofenadine, loratadine, hydroxyzine, doxylamine, xylazine
**Anti-psychotics**	Risperidone, quetiapine, olanzapine, aripiprazole, clozapine, haloperidol, chlorpromazine, ziprasidone, paliperidone, trifluoperazine, perphenazine, fluphenazine, lurasidone, pimozide
**Barbiturates**	Butalbital, phenobarbital , pentobarbital, butabarbital, amobarbital
**Benzodiazepines**	Benzodiazapene, etizolam, chlordiazepoxide, lorazepam, flubromazolam, nordiazepam, diazepam, pyrazolam, clonazepam, estazolam, xanax, alprazolam, flualprazolam
**Cocaine**	Cocaine, cocaethylene
**Fentanyl**	Fentanyl, 4-ANPP, carfentanil, acetylfentanyl
**Hallucinogens**	Phencycldine, LSD, diethylamide, ketamine, PCP, methylenedioxyamphetamine
**Heroin**	Heroin
**MDMA**	3,4-methylenedioxymethamphetamine, MDMA, methylenedioxymethamphetamine, 3,4-methylenedioxymethaphetamine
**MDA**	Methylenedioxyamphetamine, methylenedioxyamphetamine , MDA
**Methamphetamine**	Methamphetamine
**Muscle relaxants**	Cyclobenzaprine, baclofen, carisoprodol, metaxalone, methocarbamol, tizanidine, orphenadrine
**Prescription opioids**	Hydrocodone, oxycodone, hydromorphone, oxymorphone, codeine, oxycontin, methadone, percocet, buprenorphine, meperidine, morphine, tapentadol, tramadol, naltrexone, levorphanol

eTable 2. Co-occurrence of substances involved in overdose deaths

Substance	Heroin	Fentanyl	Prescription opioids	Methamphetamine	Cocaine	Benzodiazepines	Alcohol	Others
Heroin	1,613	1,133	268	314	441	264	315	538
Fentanyl	1,133	4,758	736	724	1,478	788	1,070	1,501
Prescription opioids	268	736	1,197	120	256	363	229	491
Methamphetamine	314	724	120	1,876	193	85	131	1,876
Cocaine	441	1,478	256	193	2,247	266	551	518
Benzodiazepines	264	788	363	85	266	1,076	272	505
Alcohol	315	1,070	229	131	551	272	2,866	443
Others	538	1,501	491	1,876	518	505	443	3,019

© 2022 Goodman-Meza D et al. *JAMA Network Open*.

eTable 3. Bootstrapped diagnostic metrics and 95% confidence intervals of best performing models in test dataset (n = 7,087) using TF-IDF as feature representations.

Metric	Any Opioids	Heroin	Fentanyl	Prescription Opioids	Methamphetamine	Cocaine	Benzodiazepines	Alcohol	Others
**F-score**	0.969	1.00	0.999	0.308	0.992	0.999	0.771	0.968	0.777
	(0.959-0.979)	(1.00-1.00)	(0.998-1.00)	(0.211-0.468)	(0.979-0.997)	(0.997-1.00)	(0.716-0.826)	(0.953-0.980)	(0.743-0.808)
**Accuracy**	0.990	1.00	1.00	0.964	0.999	1.00	0.986	0.995	0.967
	(0.987-0.993)	(1.00-1.00)	(1.00-1.00)	(0.958-0.974)	(0.998-1.00)	(1.00-1.00)	(0.983-0.99)	(0.992-0.997)	(0.962 - 0.972)
**Kappa**	0.963	1.00	0.999	0.290	0.991	0.999	0.764	0.965	0.76
	(0.951-0.974)	(1.00-1.00)	(0.998-1.00)	(0.193-0.455)	(0.978-0.996)	(0.996-1.00)	(0.708-0.821)	(0.949-0.978)	(0.723-0.792)
**Sensitivity (Recall)**	0.960	1.00	0.999	0.262	0.995	0.999	0.699	0.952	0.708
	(0.944-0.976)	(1.00-1.00)	(0.997-1.00)	(0.172-0.408)	(0.986-1.00)	(0.993-1.00)	(0.617-0.776)	(0.927-0.973)	(0.656-0.760)
**Specificity**	0.996	1.00	1.00	0.987	0.999	1.00	0.996	0.999	0.99
	(0.993-0.998)	(1.00-1.00)	(1.00-1.00)	(0.981-0.992)	(0.998-1.00)	(1.00-1.00)	(0.993-0.998)	(0.997-1.00)	(0.985-0.995)
**Positive predictive value (Precision)**	0.977	1.00	1.00	0.381	0.989	1.00	0.864	0.984	0.864
	(0.961-0.991)	(1.00-1.00)	(1.00-1.00)	(0.261-0.568)	(0.965-1.00)	(1.00-1.00)	(0.776-0.946)	(0.966-1.00)	(0.805-0.918)
**Negative predictive value**	0.993	1.00	1.00	0.977	1.00	1.00	0.990	0.996	0.974
	(0.99-0.995)	(1.00-1.00)	(1.00-1.00)	(0.972-0.983)	(0.999-1.00)	(1.00-1.00)	(0.986-0.993)	(0.993-0.998)	(0.969-0.980)
**AUROC**	0.998	1.00	1.00	0.893	0.997	1.00	0.981	0.991	0.98
	(0.996-0.999)	(1.00-1.00)	(1.00-1.00)	(0.851-0.931)	(0.993-1.00)	(0.994-1.00)	(0.97-0.99)	(0.984-0.998)	(0.976-0.985)

Values are means of 1,000 resamples bootstrapping procedure, values in parenthesis are lower and upper bounds of 95% percentiles for the bootstrapping procedure.

eTable 4. Bootstrapped diagnostic metrics and 95% confidence intervals of best performing models in test dataset (n = 7,087) using word embedding (GloVe) as feature representations.

Metric	Any Opioids	Heroin	Fentanyl	Prescription Opioids	Methamphetamine	Cocaine	Benzodiazepines	Alcohol	Others
**F-score**	0.966	1.00	0.999	0.378	0.998	0.999	0.525	0.942	0.750
	(0.956 - 0.976)	(1.00 - 1.00)	(0.999 – 1.00)	(0.205 - 0.537)	(0.993 – 1.00)	(0.997 – 1.00)	(0.320 - 0.612)	(0.924 - 0.960)	0.715 - 0.785
**Accuracy**	0.989	1.00	1.00	0.968	1.00	1.00	0.975	0.991	0.961
	(0.985 - 0.992)	(1.00 - 1.00)	(1.00 - 1.00)	(0.959 - 0.977)	(0.999 – 1.00)	(1.00 - 1.00)	(0.966 - 0.980)	(0.988 - 0.994)	0.955 - 0.966
**Kappa**	0.96	1.00	0.999	0.363	0.997	0.999	0.512	0.938	0.727
	(0.947 - 0.971)	(1.00 - 1.00)	(0.998 – 1)	(0.189 - 0.525)	(0.992 – 1.00)	(0.997 – 1.00)	(0.289 - 0.601)	(0.918 - 0.956)	0.691 - 0.767
**Sensitivity (Recall)**	0.957	1.00	0.999	0.296	0.995	0.999	0.501	0.926	0.718
	(0.94 - 0.974)	(1.00 - 1.00)	(0.997 – 1.00)	(0.145 - 0.448)	(0.985 – 1.00)	(0.994 – 1.00)	(0.286 - 0.608)	(0.897 - 0.952)	0.665 - 0.77
**Specificity**	0.995	1.00	1.00	0.991	1.00	1.00	0.989	0.997	0.982
	(0.992 - 0.999)	(1.00 - 1.00)	(1.00 - 1.00)	(0.986 - 0.996)	(1.00 - 1.00)	(1.00 - 1.00)	(0.983 - 0.994)	(0.994 - 0.999)	0.977 - 0.988
**Positive predictive value (Precision)**	0.976	0.999	1.00	0.537	1.00	0.999	0.56	0.96	0.786
	(0.956 - 0.993)	(1.00 - 1.00)	(1.00 - 1.00)	(0.319 - 0.756)	(1.00 - 1.00)	(0.994 – 1.00)	(0.387 - 0.698)	(0.933 - 0.984)	0.729 - 0.843
**Negative predictive value**	0.992	1.00	1.00	0.976	1.00	1.00	0.986	0.993	0.975
	(0.988 - 0.995)	(1.00 - 1.00)	(1.00 - 1.00)	(0.969 - 0.983)	(0.999 – 1.00)	(1.00 - 1.00)	(0.978 - 0.990)	(0.991 - 0.996)	0.97 - 0.98
**AUROC**	0.997	1.00	1.00	0.948	0.998	1.00	0.936	0.983	0.974
	(0.994 - 0.999)	(1.00 - 1.00)	(1.00 - 1.00)	(0.932 - 0.963)	(0.993 – 1.00)	(0.994 – 1.00)	(0.900 - 0.966)	(0.972 - 0.993)	0.966 - 0.981

Values are means of 1,000 resamples bootstrapping procedure, values in parenthesis are lower and upper bounds of 95% percentiles for the bootstrapping procedure.

GloVe with 6 billion tokens and 100 dimensions was used in this analysis.

© 2022 Goodman-Meza D et al. *JAMA Network Open*.

eTable 5. Confusion matrix for any opioids

		TF-IDF		Word embeddings (GloVe)		CUI embeddings (CUI2vec)	
Predicted	Positive	1099	11	1095	6	1128	0
	Negative	49	5928	53	5933	20	5939
		Positive	Negative	Positive	Negative	Positive	Negative
		Truth		Truth		Truth	

eTable 6. Confusion matrix for heroin

		TF-IDF		Word embeddings (GloVe)		CUI embeddings (CUI2vec)	
Predicted	Positive	326	0	326	0	326	0
	Negative	0	6761	0	6761	0	6761
		Positive	Negative	Positive	Negative	Positive	Negative
		Truth		Truth		Truth	

eTable 7. Confusion matrix for fentanyl

		TF-IDF		Word embeddings (GloVe)		CUI embeddings (CUI2vec)	
Predicted	Positive	951	0	951	0	951	0
	Negative	1	6135	1	6135	1	6135
		Positive	Negative	Positive	Negative	Positive	Negative
		Truth		Truth		Truth	

eTable 8. Confusion matrix for prescription opioids

		TF-IDF		Word embeddings (GloVe)		CUI embeddings (CUI2vec)	
Predicted	Positive	94	19	89	15	235	0
	Negative	142	6832	147	6836	1	6851
		Positive	Negative	Positive	Negative	Positive	Negative
		Truth		Truth		Truth	

© 2022 Goodman-Meza D et al. *JAMA Network Open*.

eTable 9. Confusion matrix for methamphetamine

		TF-IDF		Word embeddings (GloVe)		CUI embeddings (CUI2vec)	
Predicted	Positive	369	0	369	0	369	1
	Negative	0	6718	0	6718	0	6717
		Positive	Negative	Positive	Negative	Positive	Negative
		Truth		Truth		Truth	

eTable 10. Confusion matrix for cocaine

		TF-IDF		Word embeddings (GloVe)		CUI embeddings (CUI2vec)	
Predicted	Positive	455	0	455	0	455	0
	Negative	0	6632	0	6632	0	6632
		Positive	Negative	Positive	Negative	Positive	Negative
		Truth		Truth		Truth	

eTable 11. Confusion matrix for benzodiazepines

		TF-IDF		Word embeddings (GloVe)		CUI embeddings (CUI2vec)	
Predicted	Positive	103	13	100	15	166	0
	Negative	96	6875	99	6873	33	6888
		Positive	Negative	Positive	Negative	Positive	Negative
		Truth		Truth		Truth	

eTable 12. Confusion matrix for alcohol

		TF-IDF		Word embeddings (GloVe)		CUI embeddings (CUI2vec)	
Predicted	Positive	545	5	539	18	440	0
	Negative	29	6508	35	6495	134	6513
		Positive	Negative	Positive	Negative	Positive	Negative
		Truth		Truth		Truth	

eTable 13. Confusion matrix for others

		TF-IDF		Word embeddings (GloVe)		CUI embeddings (CUI2vec)	

© 2022 Goodman-Meza D et al. *JAMA Network Open*.

Predicted	Positive	428	75	448	85	546	1
	Negative	150	6434	130	6424	32	6508
		Positive	Negative	Positive	Negative	Positive	Negative
		Truth		Truth		Truth	

eTable 14. Error analysis for any opioids.

TF-IDF			GloVe			*CUI2vec*		
**Classification**	**n**	**%**	**Classification**	**n**	**%**	**Classification**	**n**	**%**
Missed oxycodone	15	25.0	Misinterpreted other drugs as opioid	15	22.1	Missed opioid	19	95
Missed morphine	12	20.0	Missed oxycodone	15	22.1	Missed oxycontin	1	5
Missed classified as any opioid	11	18.3	Missed morphine	11	16.2			
Missed hydrocodone	9	15.0	Missed hydrocodone	8	11.8			
Missed opioid	7	11.7	Missed opioid	8	11.8			
Missed tramadol	2	3.3	Missed tramadol	2	2.9			
Missed codeine	1	1.7	Missed buprenorphine	1	1.5			
Missed hydrocodone and hydromorphone	1	1.7	Missed codeine	1	1.5			
Missed hydrocodone and morphine	1	1.7	Missed hydrocodone, dihydrocodeine	1	1.5			
Missed oxycontin	1	1.7	Missed hydrocodone, hydromorphone	1	1.5			
			Missed hydrocodone, oxycodone	1	1.5			
			Missed hydrocodone, tramadol	1	1.5			
			Missed methadone, morphine	1	1.5			
			Missed morphine, hydrocodone	1	1.5			
			Missed oxycontin	1	1.5			

© 2022 Goodman-Meza D et al. *JAMA Network Open*.

eTable 15. Error analysis for fentanyl.

TF-IDF			GloVe			*CUI2vec*		
**Classification**	**n**	**%**	**Classification**	**n**	**%**	**Classification**	**n**	**%**
Missed carfentanil	1	100	Missed carfentanil	1	100	Missed carfentanil	1	100

eTable 16. Error analysis for prescription opioids.

TF-IDF			GloVe			*CUI2vec*		
**Classification**	**n**	**%**	**Classification**	**n**	**%**	**Classification**	**n**	**%**
Missed oxycodone	34	21.1	Missed oxycodone	34	21	Missed tramadol	1	100
Missed morphine	29	18.0	Missed tramadol	34	21			
Missed tramadol	27	16.8	Missed morphine	33	20.4			
Missed hydrocodone	23	14.2	Missed hydrocodone	20	12.3			
Misinterpreted as prescription opioid	19	11.8	Misclassified as prescription opioids	15	9.3			
Missed buprenorphine	13	8.1	Missed buprenorphine	11	6.8			
Missed tramadol and buprenorphine	4	2.5	Missed codeine	3	1.9			
Missed codeine	3	1.9	Missed hydromorphone	3	1.9			
Missed hydromorphone	3	1.9	Missed hydrocodone and oxycodone	2	1.2			
Missed hydrocodone and tramadol	1	0.6	Missed morphine and oxycodone	2	1.2			
Missed hydrocodone and oxycodone	1	0.6	Missed buprenoprhine	1	0.6			
Missed morphine and buprenorphine and tramadol	1	0.6	Missed buprenorphine and tramadol	1	0.6			
Missed morphine and hydrocodone	1	0.6	Missed hydrocodone and morphine	1	0.6			
Missed morphine and oxycodone	1	0.6	Missed hydrodocone and hydromoprhone	1	0.6			
Missed oxycodone and tramadol	1	0.6	Missed morphine and hydrocodone	1	0.6			

© 2022 Goodman-Meza D et al. *JAMA Network Open*.

eTable 17. Error analysis for benzodiazepines.

TF-IDF			GloVe			*CUI2vec*		
**Classification**	**n**	**%**	**Classification**	**n**	**%**	**Classification**	**n**	**%**
Missed clonazepam	31	28.4	Missed clonazepam	28	24.6	Missed flualprazolam	24	72.7
Missed diazepam	19	17.4	Missed flualprazolam	20	17.5	Missed etizolam	7	21.2
Missed flualprazolam	16	14.7	Missed diazepam	19	16.7	Missed flubromazolam	1	3.0
Misinterpreted other drugs for benzodiazepine	13	11.9	Misclassified as benzodiazepine	15	13.2	Missed pyrazolam	1	3.0
Missed lorazepam	11	10.1	Missed lorazepam	11	9.6	Missed flualprazolam	24	72.7
Missed etizolam	7	6.4	Missed etizolam	7	6.1			
Missed nordiazepam	6	5.5	Missed nordiazepam	6	5.3			
Missed clonazepam, flualprazolam	1	0.9	Missed flubromazolam and clonazepam	2	1.8			
Missed flualprazolam and etizolam	1	0.9	Missed alprazolam	1	0.9			
Missed flubromazolam	1	0.9	Missed chlordiazepoxide	1	0.9			
Missed pyrazolam	1	0.9	Missed clonazepam and flualprazolam	1	0.9			
Missed temazepam	1	0.9	Missed demoxepam	1	0.9			
Missed chlordiazepoxide	1	0.9	Missed pyrazolam	1	0.9			
			Missed temazepam	1	0.9			

© 2022 Goodman-Meza D et al. *JAMA Network Open*.

eTable 18. Error analysis for alcohol.

TF-IDF			GloVe			*CUI2vec*		
**Classification**	**n**	**%**	**Classification**	**n**	**%**	**Classification**	**n**	**%**
Missed alcoholic	15	44.1	Misclassified as alcohol	18	34	Missed ethanolism	65	48.5
Missed alcoholisim	11	32.4	Missed alcoholic	17	32.1	Missed alcoholism	51	38.1
Misclassified as alcohol	5	14.7	Missed alcoholism	6	11.3	Missed alcoholic	15	11.2
Coding error	3	8.8	Missed ethanolism	6	11.3	Coding error	3	2.2
			Coding error	3	5.7	Missed ethanolism	65	48.5
			Missed alcohol	2	3.8			
			Missed alcohol use disorder	1	1.9			

© 2022 Goodman-Meza D et al. *JAMA Network Open*.

eTable 19. Error analysis for “other” substances.

TF-IDF			GloVe			*CUI2vec*		
**Classification**	**n**	**%**	**Classification**	**n**	**%**	**Classification**	**n**	**%**
Misclassified as "others"	75	33.3	Misclassified as others	85	39.5	Missed xylazine	18	54.5
Missed gabapentin	16	7.1	Missed gabapentin	20	9.3	Missed MDMA	3	9.1
Missed amphetamine	13	5.8	Missed amphetamine	13	6	Missed duloxetine	2	6.1
Missed MDMA	10	4.4	Missed xylazine	12	5.6	Missed olanzapine	2	6.1
Missed diazepam as anticonvulsant	8	3.6	Coding error	11	5.1	Missed pentobarbital	2	6.1
Missed xylazine	7	3.1	Missed mdma	10	4.7	Misclassified as other	1	3.0
Missed citalopram	5	2.2	Missed citalopram	6	2.8	Missed clozapine	1	3.0
Missed diazepine as anticonvulsant	4	1.8	Missed hydroxizine	5	2.3	Missed LSD	1	3.0
Missed quetiapine	4	1.8	Missed venlafaxine	3	1.4	Missed metaxalone	1	3.0
Missed venlafaxine	4	1.8	Missed doxepin	2	0.9	Missed orphenadrine	1	3.0
Missed cyclobenzaprine	3	1.3	Missed duloxetine	2	0.9	Missed pregabalin and diphenhydramine	1	3.0
Missed gabapentin and cyclobenzaprine	2	0.9	Missed lamotrigine	2	0.9			
Missed gabapentin and xylazine	2	0.9	Missed olanzapine	2	0.9			
Missed gabapentin, phenobarbital and diphenhydramine	2	0.9	Missed paroxetine	2	0.9			
Missed hydroxyzine	2	0.9	Missed quetiapine	2	0.9			
Missed lamotrigine	2	0.9	Missed sertraline	2	0.9			
Missed olanzapine	2	0.9	Missed trazadone	2	0.9			
Missed pentobarbital	2	0.9	Missed zolpidem	2	0.9			

© 2022 Goodman-Meza D et al. *JAMA Network Open*.

Missed sertraline	2	0.9	Other errors with no more than one mention	4					
Other errors with no more than one mention	60								

eFigure 1: Variable importance plot for predicting category "Any opioids".

Coefficients were extracted using TF-IDF and logistic regression. Tokens in the Positive (right) plot increase the probability that the text description will be classified to the substance. Tokens in the Negative (left) plot decrease the probability that the text description will be classified to the substance.

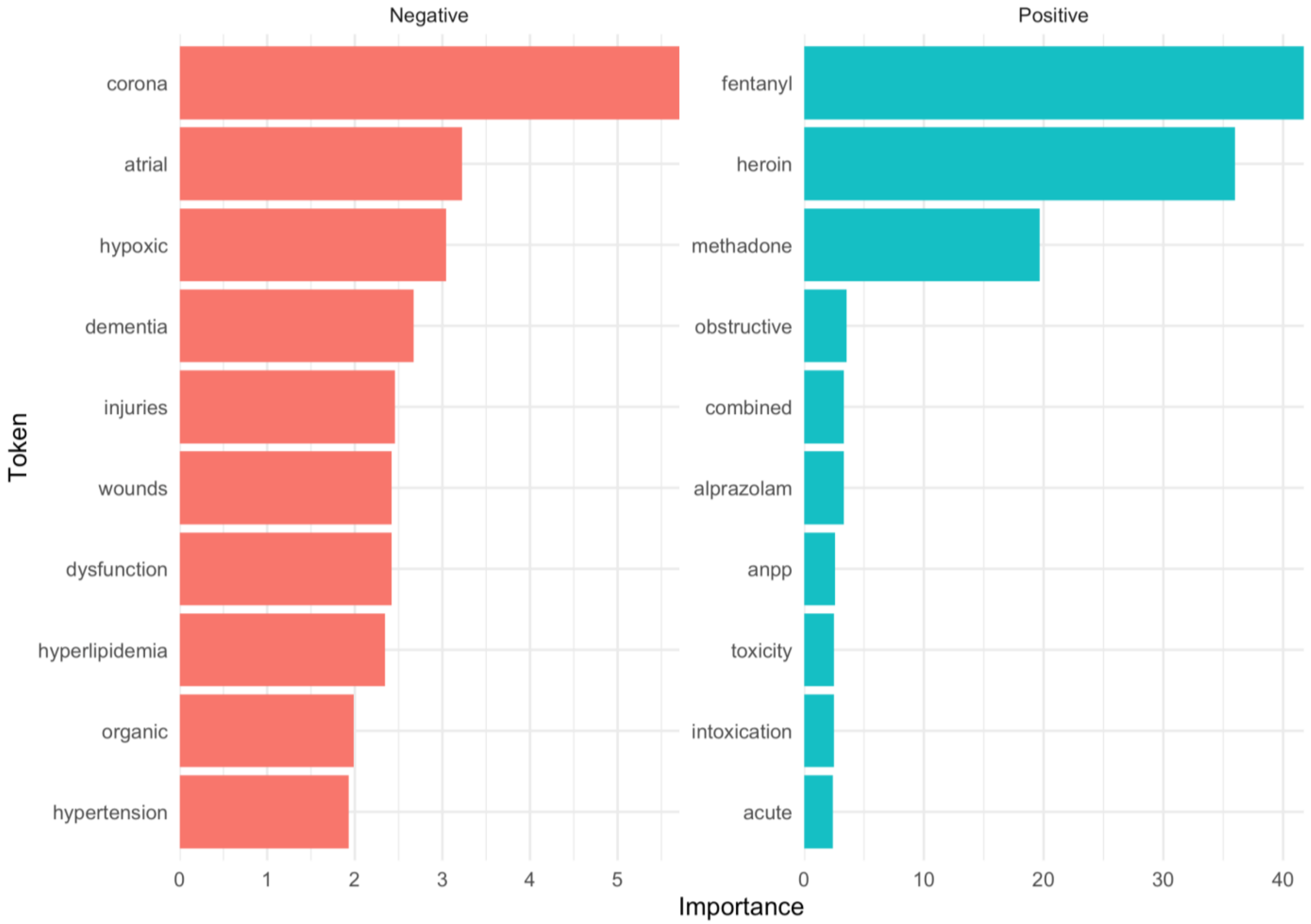

eFigure 2: Variable importance plot for predicting category “Heroin”.

Coefficients were extracted using TF-IDF and logistic regression.

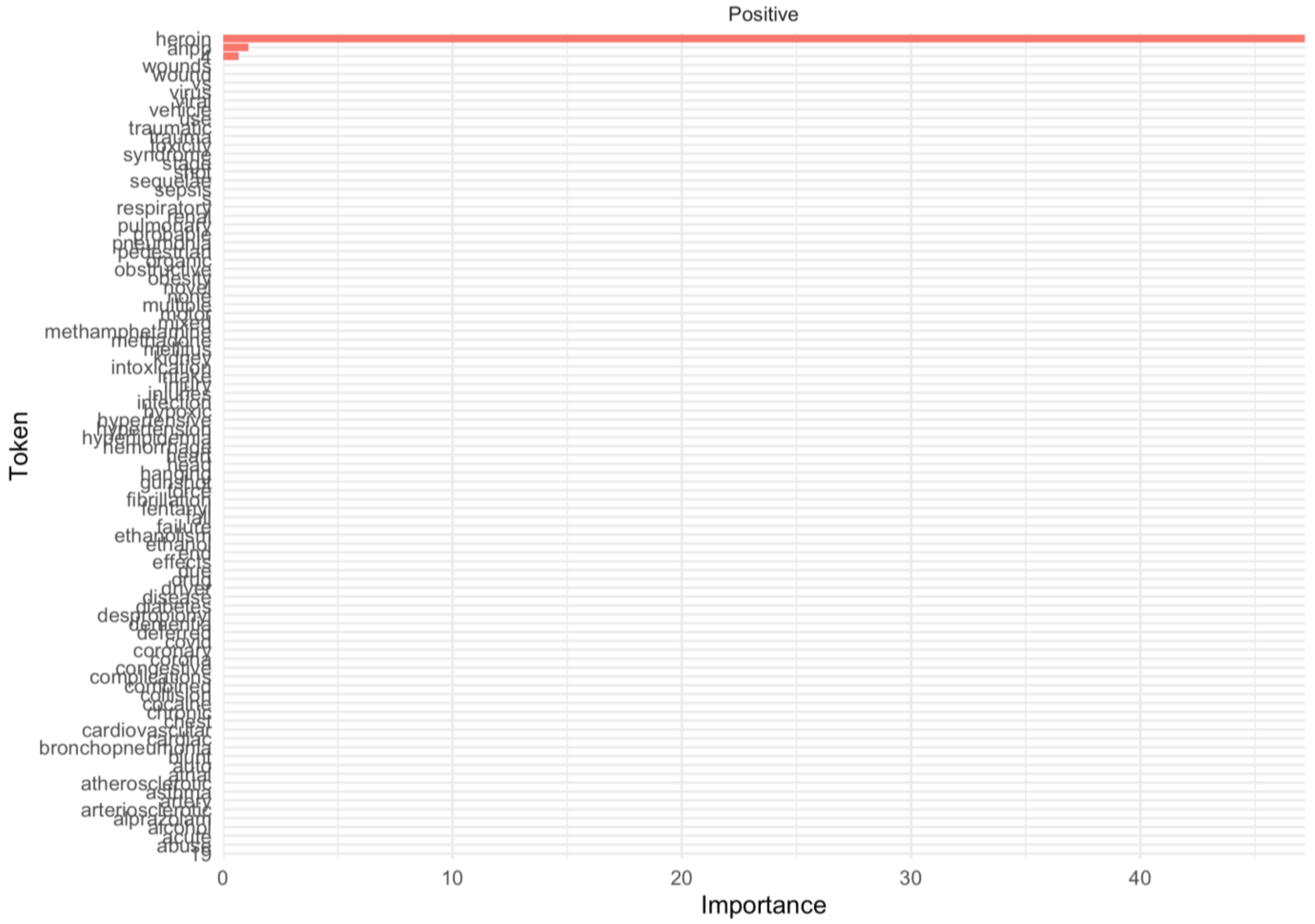

eFigure 3: Variable importance plot for predicting category “Fentanyl”.

Coefficients were extracted using TF-IDF and logistic regression.

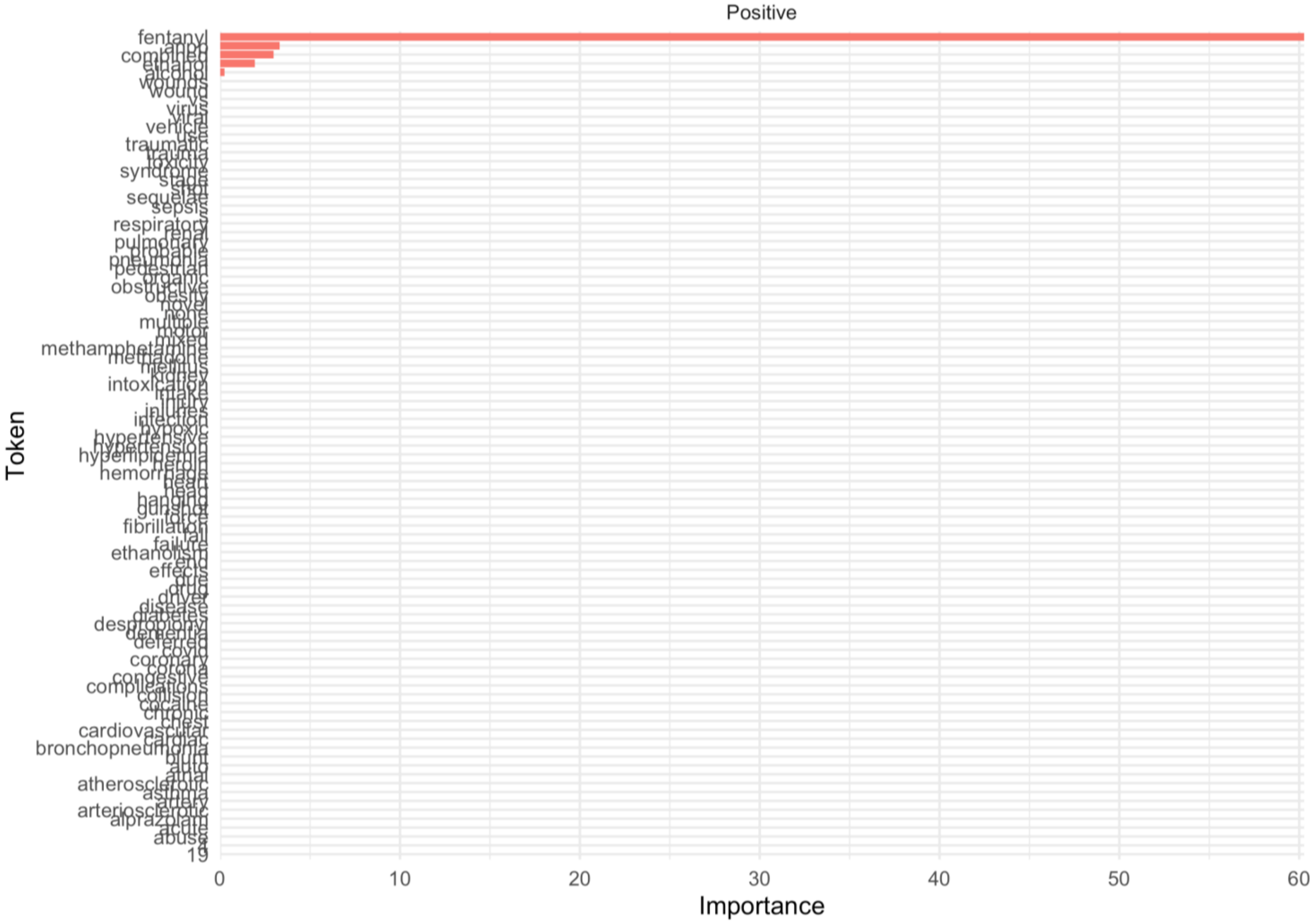

© 2022 Goodman-Meza D et al. *JAMA Network Open*.

eFigure 4: Variable importance plot for predicting category “Prescription opioids”.

Coefficients were extracted using TF-IDF and logistic regression. Tokens in the Positive (right) plot increase the probability that the text description will be classified to the substance. Tokens in the Negative (left) plot decrease the probability that the text description will be classified to the substance.

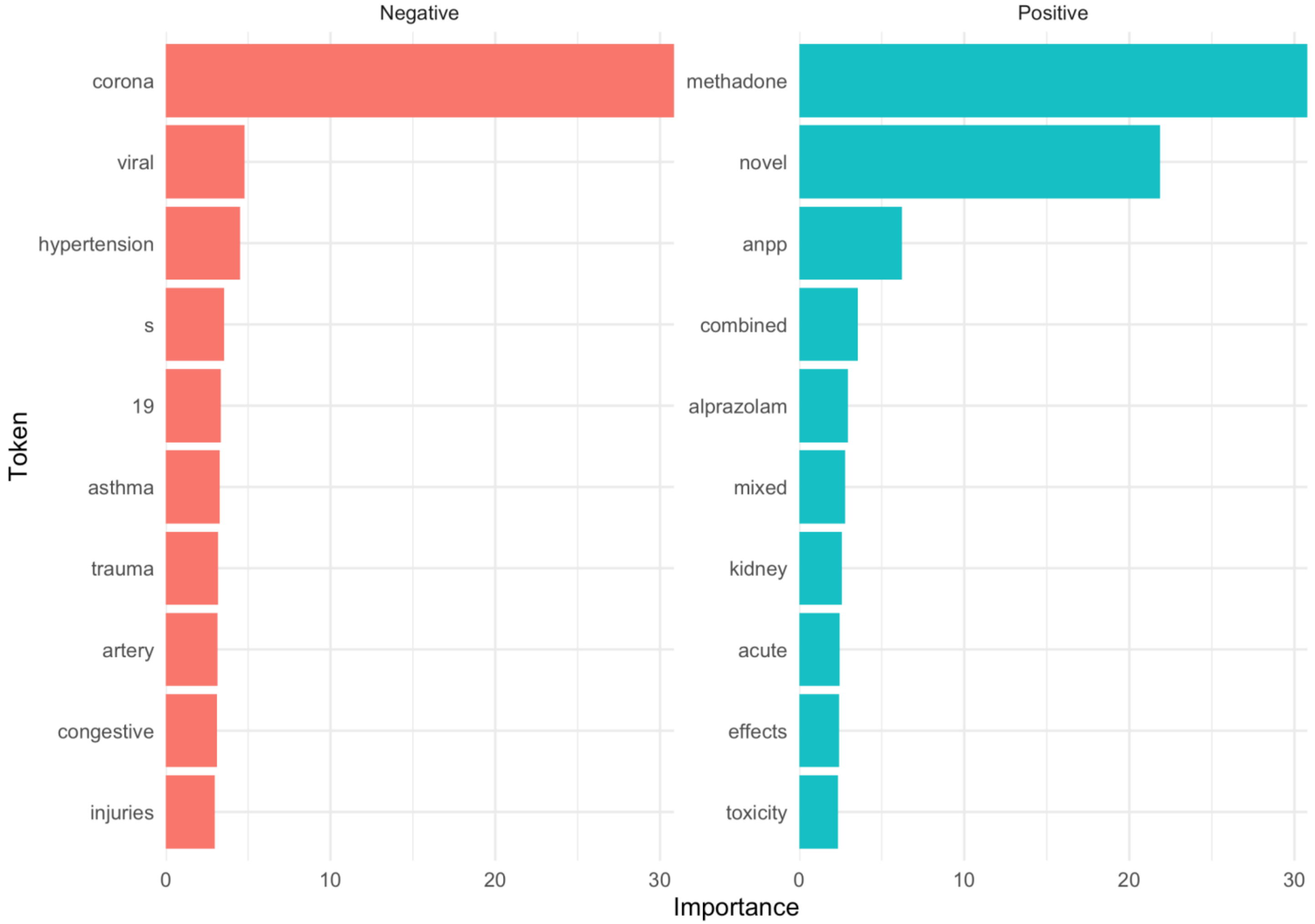

© 2022 Goodman-Meza D et al. *JAMA Network Open*.

eFigure 5: Variable importance plot for predicting category “Methamphetamine”.

Coefficients were extracted using TF-IDF and logistic regression.

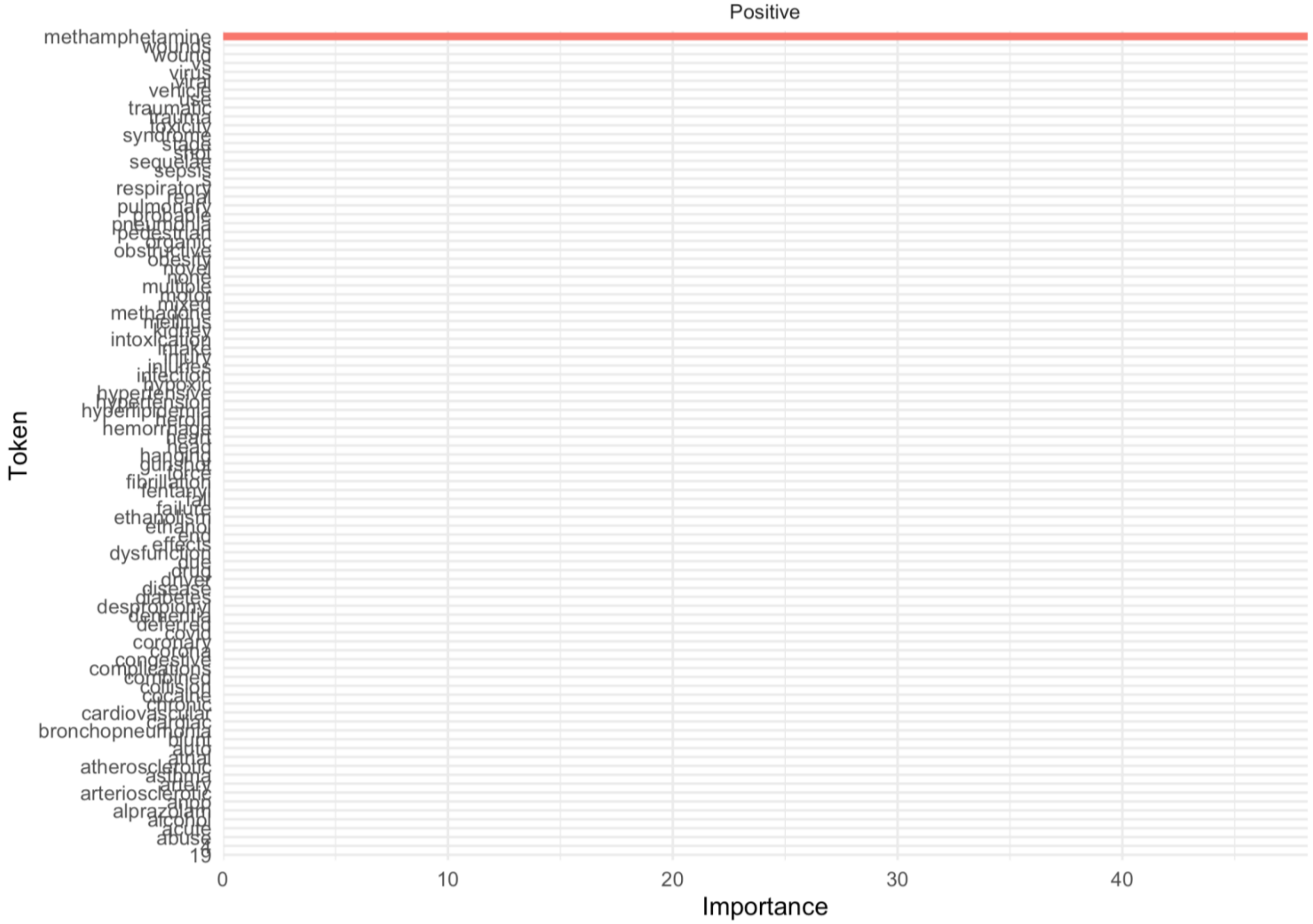

eFigure 6: Variable importance plot for predicting category “Cocaine”.

Coefficients were extracted using TF-IDF and logistic regression.

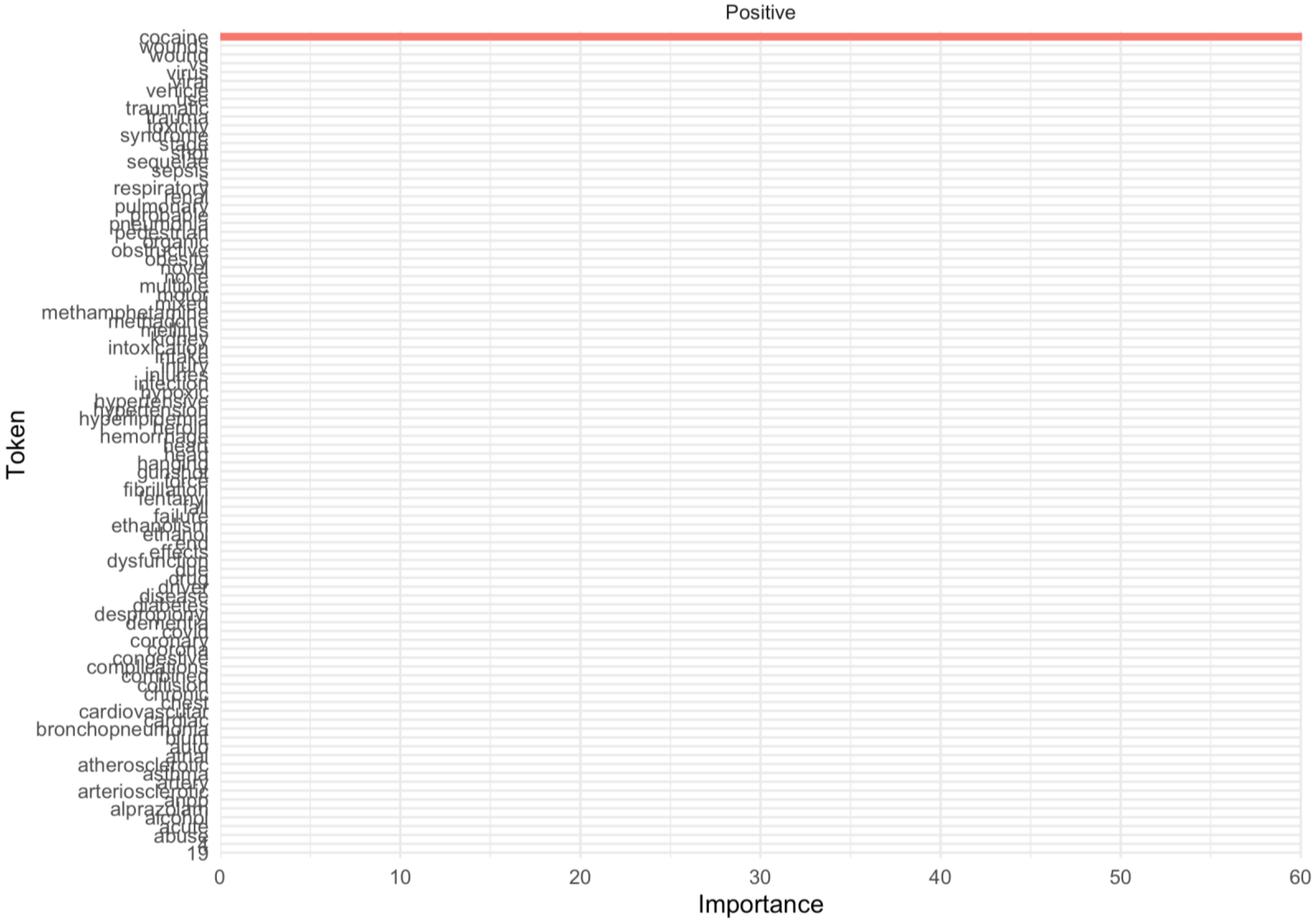

© 2022 Goodman-Meza D et al. *JAMA Network Open*.

eFigure 7: Variable importance plot for predicting category “Benzodiazepines”.

Coefficients were extracted using TF-IDF and logistic regression. Tokens in the Positive (right) plot increase the probability that the text description will be classified to the substance. Tokens in the Negative (left) plot decrease the probability that the text description will be classified to the substance.

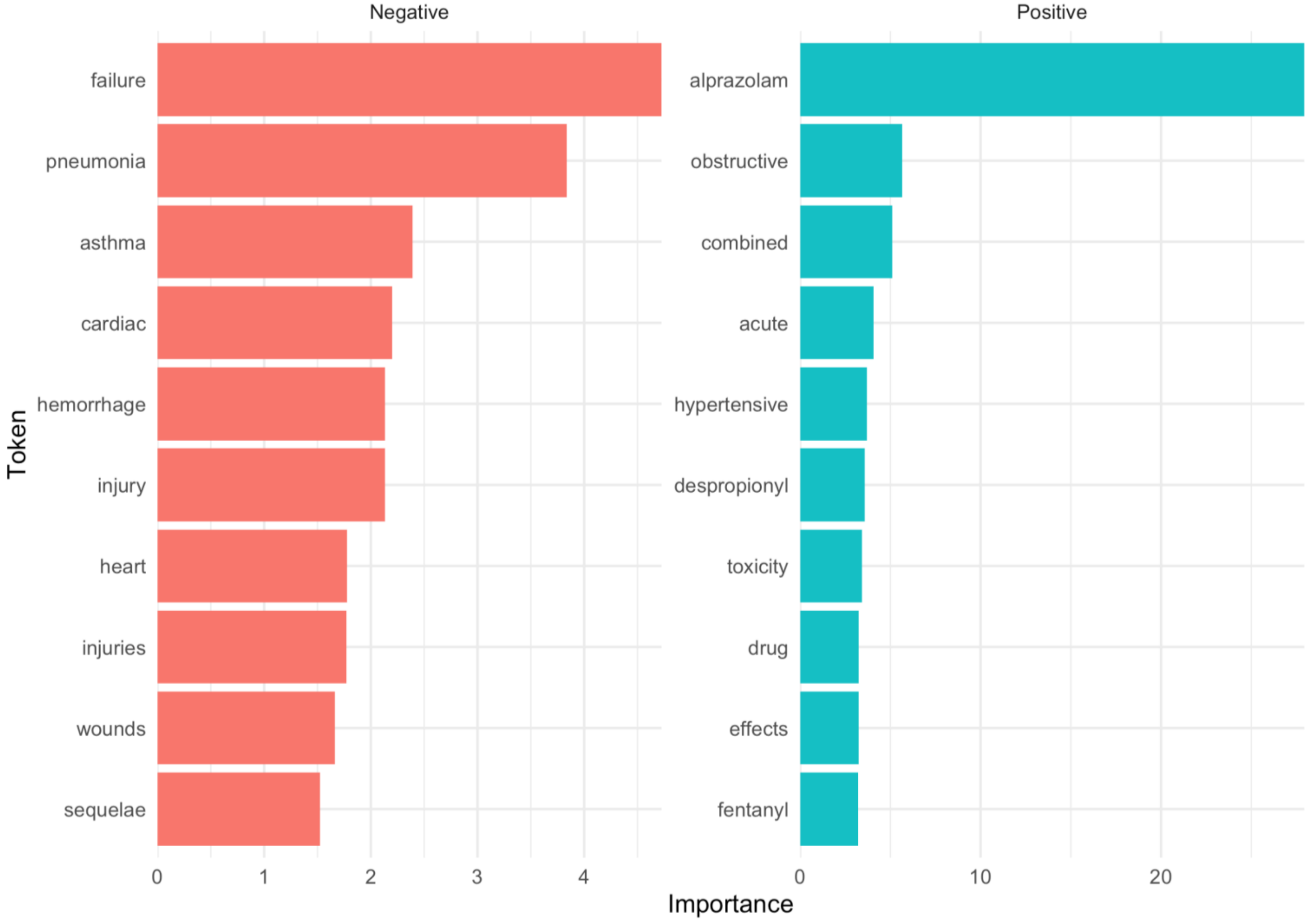

eFigure 8: Variable importance plot for predicting category “Alcohol”.

Coefficients were extracted using TF-IDF and logistic regression. Tokens in the Positive (right) plot increase the probability that the text description will be classified to the substance. Tokens in the Negative (left) plot decrease the probability that the text description will be classified to the substance.

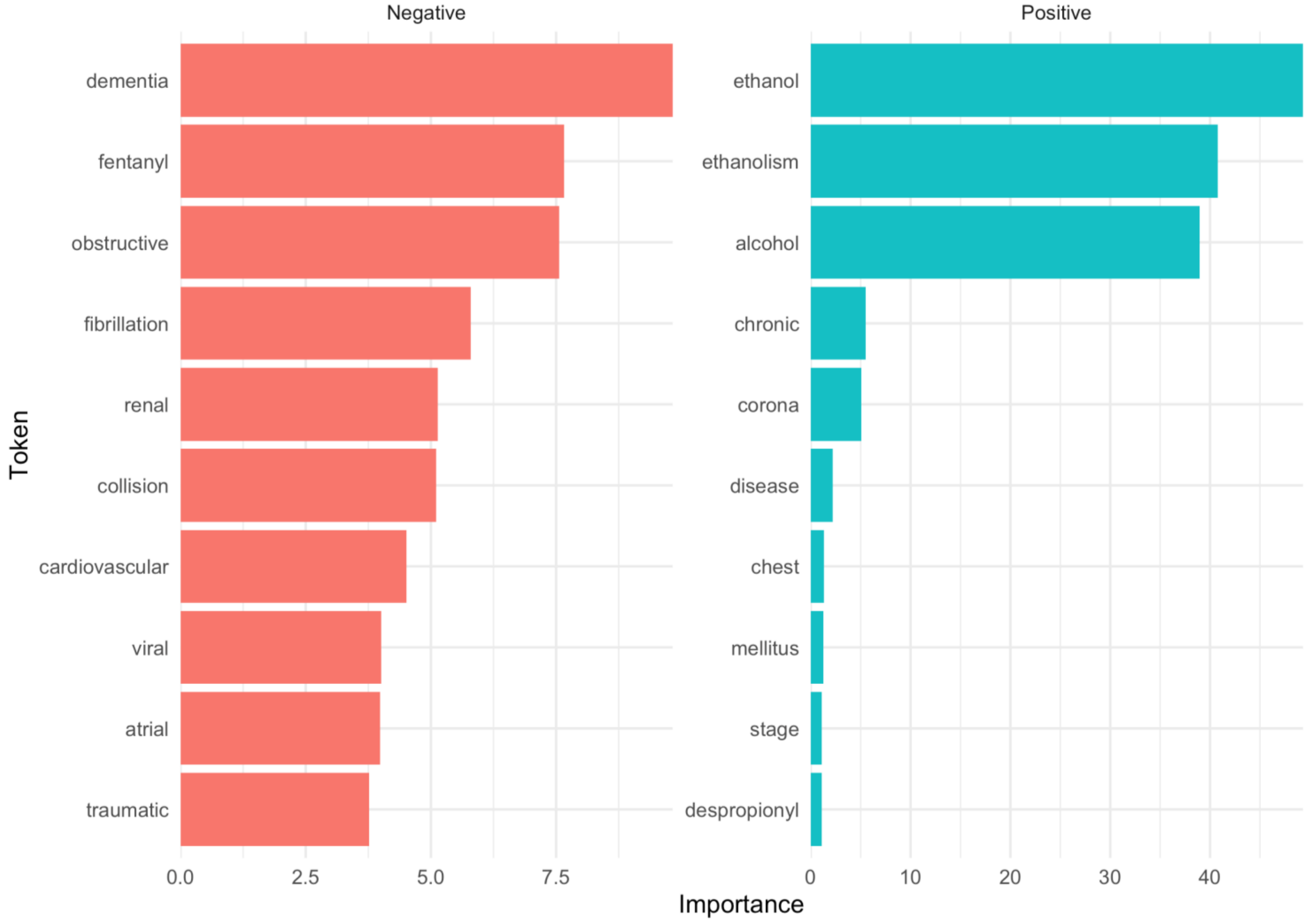

© 2022 Goodman-Meza D et al. *JAMA Network Open*.

eFigure 9: Variable importance plot for predicting category “Others”.

Coefficients were extracted using TF-IDF and logistic regression. Tokens in the Positive (right) plot increase the probability that the text description will be classified to the substance. Tokens in the Negative (left) plot decrease the probability that the text description will be classified to the substance.

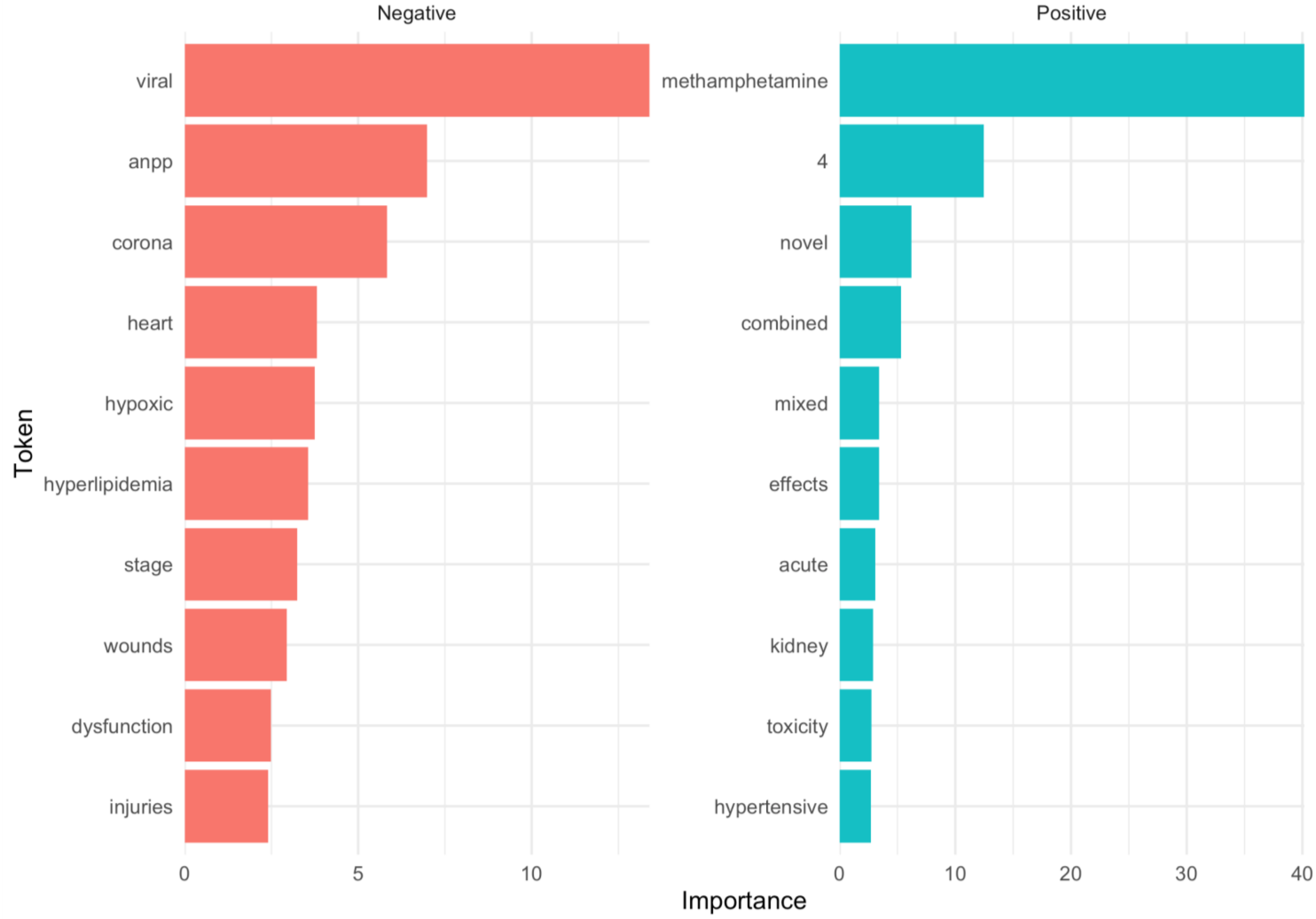

Supplement: Supplement. — eTable 1. Classifications and Keywords of Substances Related to Overdoses eTable 2. Co-occurrence of Substances Involved in Overdose Deaths eTable 3. Bootstrapped diagnostic Metrics and 95% Confidence Intervals of Best Performing Models in Test Dataset (N=7,087) Using TF-IDF as Feature Representations eTable 4. Bootstrapped Diagnostic Metrics and 95% Confidence Intervals of Best Performing Models in Test Dataset (N=7,087) Using Word Embedding (GloVe) as Feature Representations eTable 5. Confusion Matrix for Any Opioids eTable 6. Confusion Matrix for Heroin eTable 7. Confusion Matrix for Fentanyl eTable 8. Confusion Matrix for Prescription Opioids eTable 9. Confusion Matrix for Methamphetamine eTable 10. Confusion Matrix for Cocaine eTable 11. Confusion Matrix for Benzodiazepines eTable 12. Confusion Matrix for Alcohol eTable 13. Confusion Matrix for Others eTable 14. Error Analysis for Any Opioids eTable 15. Error Analysis for Fentanyl eTable 16. Error Analysis for Prescription Opioids eTable 17. Error Analysis for Benzodiazepines eTable 18. Error Analysis for Alcohol eTable 19. Error Analysis for “Other” Substances eFigure 1. Variable Importance Plot for Predicting Category “Any Opioids” eFigure 2. Variable Importance Plot for Predicting Category “Heroin” eFigure 3. Variable Importance Plot for Predicting Category “Fentanyl” eFigure 4. Variable Importance Plot for Predicting Category “Prescription Opioids” eFigure 5. Variable Importance Plot for Predicting Category “Methamphetamine” eFigure 6. Variable Importance Plot for Predicting Category “Cocaine” eFigure 7. Variable Importance Plot for Predicting Category “Benzodiazepines” eFigure 8. Variable Importance Plot for Predicting Category “Alcohol” eFigure 9. Variable Importance Plot for Predicting Category “Others” [file jamanetwopen-e2225593-s001.pdf]
